# Allogeneic stem cell transplantation without preconditioning in a child with therapy-related myelodysplastic syndrome: A case report

**DOI:** 10.1097/MD.0000000000032770

**Published:** 2023-02-10

**Authors:** Yi-Ling Tung, Yi-Lun Wang, Tsung-Yen Chang, Chia-Chi Chiu, Yu-Chuan Wen, Tang-Her Jaing

**Affiliations:** a Department of Pediatrics, Chang Gung Children’s Hospital, Chang Gung University, Taoyuan, Taiwan; b Divisions of Hematology and Oncology, Chang Gung Children’s Hospital, Chang Gung University, Taoyuan, Taiwan; c Department of Nursing, Chang Gung Memorial Hospital, Linkou, Taoyuan, Taiwan.

**Keywords:** hematopoietic stem cell transplantation, infant acute lymphoblastic leukemia, recurrent infection, therapy-related myelodysplastic syndrome

## Abstract

Infants with mixed-lineage leukemia (MLL)-rearranged leukemia are usually refractory to standard induction therapy and are not immediate candidates for allogeneic hematopoietic stem cell transplantation (allo-HSCT). Chromosome 11q23 translocations, resulting in MLL rearrangement, have been well characterized in infant acute lymphoblastic leukemia (ALL). While t(4;11) ALL continues to have carry a bleak prognosis, patients with therapy-related myelodysplastic syndrome (t-MDS) have a shorter median overall survival than those compared with de novo MDS.

**Patient concerns:**

We describe a child with t-MDS who evolved from MLL-rearranged ALL and was successfully treated with HSCT without toxic preconditioning.

**Diagnoses:**

MDS diagnosis was based on morphological characteristics of bone marrow dysplasia in patients with clinical manifestations evidence of hematopoiesis impairments by different combinations of anemia, leukopenia, neutropenia, and thrombocytopenia.

**Interventions:**

Although the best donor for allo-HSCT is generally considered an human leukocyte antigen-matched sibling, only ~ 30% of patients have a suitable sibling. HSCT from an unrelated donor is a suitable option for patients with t-MDS who do not have matched sibling donors.

**Outcomes:**

Allo-HSCT without recipient preconditioning could be a promising treatment option for t-MDS, especially for patients with recurrent or persistent infections.

**Lessons:**

Cytogenetics, prognosis, and treatment of t-MDS are briefly discussed. Preconditioning before allo-HSCT seriously damages immune function. This work reviews our experience with a patient with t-MDS following ALL complicated by recurrent infections, and highlights our choice to omit preconditioning from allo-HSCT.

## 1. Introduction

Therapy-related myelodysplastic syndrome (t-MDS) is a lethal complication of cancer treatments. A long latency period of 5 to 7 years after exposure to alkylating agents is expected prior to the development of t-MDS.^[1]^ In contrast, disorders that develop secondary to topoisomerase II inhibitors present as acute myeloid leukemia are related to balanced translocations involving chromosome bands 11q23 or 21q22.^[2–4]^ Herein, we present a case of infant acute lymphoblastic leukemia (ALL) that further evolved to t-MDS and was treated with an upfront transplant.

## 2. Case report

**Figure 1.**
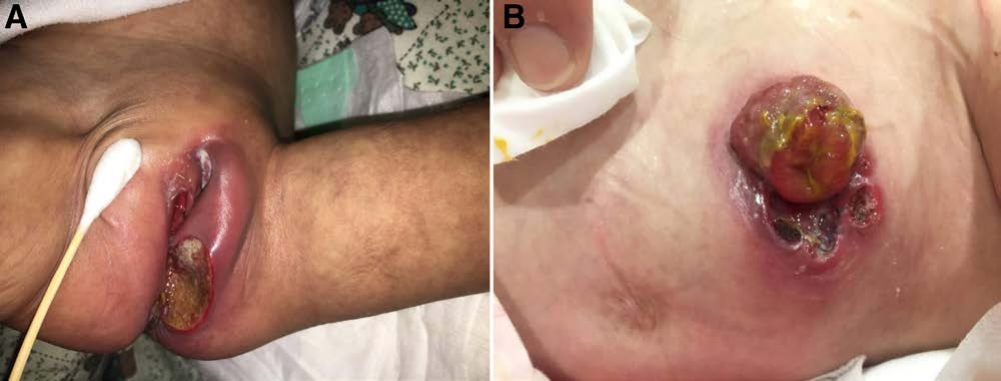
Images showing (A) worsening perineal infection and (B) wound dehiscence of the colostomy.

This 3-year-4-month-old girl was diagnosed with pro-B ALL during infancy in April 2017. Chromosome studies reported t(4;11)(q21;q23), which is compatible with mixed-lineage leukemia-AF4 fusion transcripts identified by reverse transcription-polymerase chain reaction studies and enrolled in the very high-risk group. She had completed induction and consolidation chemotherapy but had aborted continuation chemotherapy since week 40 due to recurrent systemic infections. The perineal wound had deteriorated. Debridement was completed to remove the thick eschar and necrotic tissue, and colostomy was performed for stool bypass (Fig. 1). Follow-up bone marrow aspirates are characterized by marked myeloid hyperplasia and a blast count of 3%, which may evolve into myelodysplasia, associated with the risk of leukemic transformation. In the following 2 years, her medical history was rich with frequent hospital admissions for recurrent infections, including pneumonia, *Pseudomonas septicemia*, and vancomycin-resistant *enterococcus* wound infections.

Blood cultures, oxygen supplementation, and inhalation therapy were administered for repeated admissions. She received empiric antibiotics for fever and granulocytopenia of unknown etiology. Moreover, wound cultures from the colostomy still yielded vancomycin-resistant *enterococcus*, so daptomycin was substituted for teicoplanin. Granulocyte colony-stimulating factor was administered for leukopenia. Intravenous Immunoglobulin was given every 3 weeks to replace immunoglobulins. However, unremitting fever persisted, although appropriate antibiotics were prescribed. Bronchoalveolar lavage revealed destructive laryngeal mucosa and increased sticky secretion in the tracheobronchial trees. The bronchoalveolar lavage culture yielded *Actinomyces odontolyticus*. Computed tomography scan of the head and lung revealed bilateral pneumonia and paranasal sinusitis.

Analysis of MRD and mixed-lineage leukemia/AF4 rearrangements was negative. However, the percentage of blasts in the bone marrow and degree of dysplasia were higher than those in the previous aspiration. The patient was diagnosed with t-MDS secondary to ALL. While her general condition stabilized, allogeneic hematopoietic stem cell transplantation (allo-HSCT) was performed in July 2020. As no matched-related donor was available, the patient underwent allo-HSCT from a human leukocyte antigen (HLA)-mismatched unrelated donor. The HLA typing of the patient was A1101, 2407; B1301, 4001; C0304, 0401; DRB_1_1101, 1602. The HLA types of the donor were A1101, 2402; B1301, 4001; C0304, -; DRB_1_1101, 1602.

**Figure 2.**
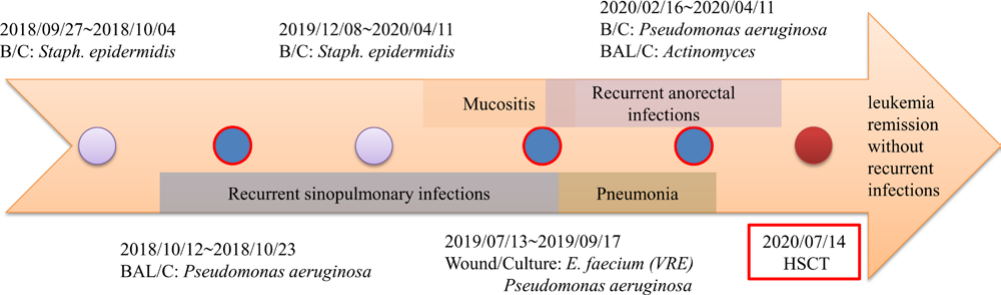
Timeline of the clinical course of this patient, showing events during recurrent infections to hematopoietic stem cell transplantation.

Due to the thorough history of the patient, we felt that a ‘classical’ preconditioning regimen would not be tolerated. No preconditioning chemotherapy was administered, and she received only cyclosporine for prophylaxis of graft-versus-host disease (GVHD). The number of infused CD34 cells was 10.12 × 10^6^/kg. She was diagnosed with chronic GVHD involving the lungs and skin 13 months after HSCT. The patient was treated with ruxolitinib, prednisolone, and phototherapy. At the time of this report, she was alive without leukemia relapse 2.5 years post-HSCT. A timeline of the clinical course is shown in Figure 2.

## 3. Discussion

Patients with t-MDS are generally considered high risk at the time of diagnosis and are commonly referred for allo-HSCT.^[5–7]^ If the donor is suitable, upfront transplantation may prolong survival in high-risk MDS patients, which may also increase the incidence of cGVHD.^[8]^ However, this patient poses unique challenges, and little is known about the ideal treatment sequence and timing. The theoretical advantages of no conditioning regimen are the absence of chemotherapy-induced toxicity and the lower incidence of GVHD; however, there is little chance for donor myeloid engraftment.^[9]^ However, concomitant systemic infections and immunocompromised status may account for recurrent wound deterioration in our patient. As a patient develops multiple infections or infections that are difficult to eradicate, this decision can be challenging.

Childhood ALL generally has a good prognosis; most children are cured and become long-term survivors.^[10]^ However, the cumulative incidence of t-MDS/t-AML ranges from 0.8 to 6.3% at 20 years of age in children treated with standard protocols for ALL.^[11,12]^ MDS, in most cases, MDS requires allo-HSCT with a more intensive conditioning regimen. Moreover, as most trials with reduced-intensity regimens have enrolled older patients and patients with comorbid conditions,^[13]^ we may rely on the immunotherapeutic effect of donor cells for complete disease eradication. The lower the conditioning intensity, the more the patient’s cure depends on the graft-versus-leukemia effect.

In this case, the goal of HSCT was to successfully cure the t-MDS. However, physicians should not overinterpret the presence of somatic mutations. Traditional techniques for HSCT rely on the production of biological ‘space’ in the recipient bone marrow compartment with myeloablative conditioning.

## 4. Conclusion

Although pre-chemotherapy before allo-HSCT can reduce the number of remaining cancer cells to a minimum and provide a prospective outcome, considering her health condition, we discontinued chemotherapy and went straight to HSCT, with sufficient immunity imperative. Gratefully, the girl remained stationary for 2.5 years.

## Acknowledgments

We want to thank the patient and family for allowing us to share their stories for educational purposes.

## Author contributions

**Conceptualization:** Tang-Her Jaing.

**Data curation:** Yi-Ling Tung.

**Investigation:** Tsung-Yen Chang.

**Methodology:** Yi-Ling Tung, Tsung-Yen Chang.

**Project administration:** Tang-Her Jaing.

**Resources:** Yu-Chuan Wen.

**Software:** Yi-Lun Wang, Chia-Chi Chiu.

**Supervision:** Chia-Chi Chiu, Yu-Chuan Wen.

**Validation:** Yi-Lun Wang, Chia-Chi Chiu, Yu-Chuan Wen.

**Visualization:** Yi-Lun Wang, Yu-Chuan Wen.

**Writing – original draft:** Chia-Chi Chiu, Tang-Her Jaing.

**Writing – review & editing:** Tang-Her Jaing.
